# Gut Microbiota Modulates Fgf21 Expression and Metabolic Phenotypes Induced by Ketogenic Diet

**DOI:** 10.3390/nu16234028

**Published:** 2024-11-25

**Authors:** Xinyi Wei, Yunxu Lu, Shangyu Hong

**Affiliations:** State Key Laboratory of Genetic Engineering, School of Life Sciences, Fudan University, Shanghai 200438, China; weixy21@m.fudan.edu.cn (X.W.); yxlu23@m.fudan.edu.cn (Y.L.)

**Keywords:** ketogenic diet, glucose metabolism, weight loss, gut microbiota, Fgf21, amino acid, valine

## Abstract

Background: The ketogenic diet (KD) is a widely used intervention for obesity and diabetes, effectively reducing body weight and blood glucose levels. However, the molecular mechanisms by which the KD influences body weight and glucose metabolism are not fully understood. While previous research has shown that the KD affects the gut microbiota, the exact role of microbiota in mediating its metabolic effects remains unclear. Methods: In this study, we used antibiotics to eliminate the gut microbiota, confirming its necessity for the KD’s impact on weight loss and glucose metabolism. We also demonstrated the significant role of FGF21 in these processes, through antibiotics intervention in *Fgf21*-deficient mice. Results: Furthermore, we revealed that the KD alters serum valine levels via the gut microbiota, which in turn regulates hepatic *Fgf21* expression and circulating FGF21 levels through the GCN2-eIF2α-ATF5 signaling pathway. Additionally, we demonstrated that valine supplementation inhibits the elevated expression of FGF21, leading to the reduced body weight and improved glucose metabolism of the KD-fed mice. Overall, we found that the gut microbiota from the KD regulates *Fgf21* transcription via the GCN2-eIF2α-ATF5 signaling pathway. ultimately affecting body weight and glucose metabolism. Conclusion: Our findings highlight a complex regulatory network linking the KD, *Fgf21* expression, and gut microbiota, offering a theoretical foundation for targeted therapies to enhance the metabolic benefits of the KD.

## 1. Introduction

The ketogenic diet (KD) is a specialized formula diet often used for weight loss, the treatment of refractory epilepsy, and metabolic disorders such as glucose transporter type I deficiency and pyruvate dehydrogenase deficiency [1]. Studies in both humans and animals indicate that the KD induces a range of metabolic-related physiological changes, such as weight loss, reduced blood glucose levels, and enhanced insulin sensitivity, although long-term adherence has been associated with issues like hyperlipidemia [2,3,4]. The potential risks of the KD should not be overlooked. Recent clinical studies have shown that KDs can raise low-density lipoprotein cholesterol (LDL-c) levels, suggesting an increased risk of cardiovascular disease [5]. Additionally, KDs have been found to significantly induce metabolic-related fatty liver disease in both humans and mice [6,7].

The molecular mechanisms by which the KD influences hepatic glucose and lipid metabolism networks are not fully understood. Nakagawa et al. discovered that a short-term KD upregulates the expression of CREB3L3 in the liver, which can bind to PPARα to regulate fatty acid oxidation and ketogenesis [8]. Our previous work demonstrated that the KD downregulates the expression of Nicotinamide *N*-methyltransferase in the liver, which regulates hepatic glucose and lipid metabolism through its product, N1-methyl-nicotinamide [9]. The KD also causes a downregulation of *Cavin1* in the liver, and *Cavin1* knockout (KO) impairs the exchange of substances between the liver and blood, leading to glucose metabolic abnormalities [10,11]. Badman et al. conducted a gene chip analysis on liver tissues from mice on a normal diet and KD, revealing that FGF21 levels in the liver increased several dozen-fold under ketogenic conditions [12]. Secreted by the liver, FGF21 plays a crucial role in regulating glucose and lipid metabolism in the liver itself, other peripheral tissues (such as adipose tissue and pancreas), and the central nervous system [13]. Elevating FGF21 levels in mice effectively suppresses diet-induced obesity and hyperglycemia [13]. More recent studies indicate that there are various transcription factors involved in the regulation of *Fgf21* expression [12,14,15]. Nonetheless, the specific molecular mechanisms through which the KD regulates hepatic *Fgf21* expression remain unclear.

Diets significantly impact the gut microbiota, playing a dominant role in shaping the host’s microbial community and forming stable, distinct gut types [16,17]. In 2018, Olson et al. reported that the KD induces changes in the gut microbiota, which subsequently affects the composition and concentration of host metabolites, resulting in decreased systemic levels of γ-glutamylated amino acids and increased levels of GABA/glutamate in the hippocampus [18]. These changes are crucial for the anticonvulsant effects of the ketogenic diet. In 2021, Li et al. compared the impacts of two common mouse KD and popular human ketogenic recipes on the gut microbiota, metabolites, and host metabolic status, suggesting that the physiological phenotypes induced by the KDs may result from alterations in the gut microbiota and its metabolites [19].

In the current study, we aimed to investigate the role of the gut microbiota in mediating the metabolic effects of the KD, particularly its impact on *Fgf21* expression and related physiological phenotypes. This work seeks to elucidate how gut microbiota-derived changes contribute to FGF21 regulation and metabolic adaptations, providing a basis for future strategies to treat metabolic diseases such as obesity and diabetes.

## 2. Materials and Methods

### 2.1. Animal Experiments

Wild-type (WT) C57BL/6J male mice (body weight above 22–24 g), aged 6–8 weeks, and *Fgf21* whole-body knockout (*Fgf21*KO) mice, were obtained from Gempharmatech Ltd. (Nanjing, China). The mice were housed in a specific pathogen-free (SPF) facility at Fudan University under a strict 12 h light/dark cycle (8:00 a.m. to 8:00 p.m.), with humidity at 45 ± 5% and temperature at 22–24 °C, having ad libitum access to food and water.

We randomly assigned the WT male C57BL/6J mice (6–8 weeks old) into three groups based on body weight, with 4–7 mice per group. One group was fed with a control diet and received pure water as a control diet group (CD group), while the other two groups were fed with a KD. One of the KD groups received pure water (KD group), and the other received a cocktail of antibiotics in their drinking water (AKD group) for 6 weeks. The male *Fgf21KO* mice (6–8 weeks old) were also randomly assigned into two groups, with 5–6 mice per group, and fed with a KD (KD_*Fgf21KO* group) and AKD (AKD_*Fgf21KO* group) for 6 weeks. Additionally, the WT male mice fed KD were injected intraperitoneally with amino acids (200 mg/kg/day) for 14 days.

The chow diet (CD: 26.7% protein *w*/*w*, 19.5% fat *w*/*w*, 53.8% carbohydrates *w*/*w*, 3.6 kcal/g) was purchased from LabDiet (Formulab Diet 5008, LabDiet, Ritchmond, IN, USA), and the ketogenic diet (KD: 8.6% protein *w*/*w*, 75.1% fat *w*/*w*, 3.2% carbohydrates *w*/*w*, 7.24 kcal/g) obtained from Bio-Serv (F3666, Bio-Serv, Flanders, NJ, USA). The mice in the antibiotics-treated group were fed with an antibiotics cocktail solution, which was selected based on its effectiveness as demonstrated in previous research [20,21,22]. The solution contained ampicillin (0.5 mg/mL) (A600064), neomycin trisulfate hydrate (0.5 mg/mL) (A610366), erythromycin (0.5 mg/mL) (A600192), gentamicin sulfate (0.5 mg/mL) (A506614), and metronidazole (0.1 mg/mL) (A600633), all purchased from Sangon Biotech (Shanghai, China). These antibiotics were dissolved in sterile distilled water and added to drinking water (replaced every other day). Food and water intake was monitored daily, and body weight were recorded biweekly. Blood samples from the mice were collected via ophthalmic venous plexus sampling for subsequent metabolomics and biochemical analyses. Following euthanasia, organs and tissues were rapidly collected and cryopreserved in liquid nitrogen and stored in an ultra-low temperature freezer at −80 °C. All experiments were conducted at the School of Life Sciences, Fudan University, adhering to ethical standards for animal research, with approval from the Animal Ethics Committee at Fudan University. All animal experimental procedures strictly adhered to the guidelines of the Laboratory Animal Management Regulations of Fudan University (ethical approval number: IDM2024010).

### 2.2. Fasting Blood Glucose and Glucose Tolerance Test

Fasting blood glucose levels were measured after a 16 h fast (from 5:00 p.m. to 9:00 a.m.) using an Accu-Chek Performa glucometer (Accu-Chek, Roche, Basel, Switzerland). For the glucose tolerance test (GTT), the mice were overnight-fasted and injected with glucose (1.5 g/kg body weight). Blood glucose levels were measured at 0, 15, 30, 60, and 120 min post the glucose intraperitoneal injection.

### 2.3. RNA Extraction, Reverse Transcription, and Real-Time Quantitative PCR

The methods for RNA extraction, reverse transcription, and real-time quantitative PCR followed the methods of our previous study [9]. Frozen mouse liver tissue (50 mg) was homogenized, mixed with 100 μL of pre-chilled chloroform, and centrifuged. The supernatant was transferred to a new centrifuge tube containing 500 μL of isopropanol, incubated on ice, and centrifuged again. The supernatant was discarded, and 500 μL of 75% ethanol was added; after gentle inversion, the sample was centrifuged again. The supernatant was removed, allowing the RNA to air-dry at room temperature before reconstituting it in 100 μL of DEPC-treated water.

The reverse transcription (RT) of RNA was performed using the HiScript III all-in-one RT SuperMix Perfect for qPCR kit (R333, Vazyme, Nanjing, China) according to the manufacturer’s instructions, yielding complementary DNA. Subsequently, a real-time quantitative PCR (qPCR) was conducted on the cDNA using Taq Pro Universal SYBR qPCR Master Mix (Q712, Vazyme), with a high-throughput full-functional fluorescence quantitative PCR system (Q7, Thermofisher Scientific, Waltham, MA, USA). Each sample was analyzed in triplicate, and the relative expression levels of mRNA were calculated using the ΔΔCT method. The specific primers of *Fgf21* (F: GTGTCAAAGCCTCTAGGTTTCTT, R: GGTACACATTGTAACCGTCCTC), *Pparα* (F: AGAGCCCCATCTGTCCTCTC, R: ACTGGTAGTCTGCAAAACCAAA), *Nupr1* (F: TGAGACGGAGCTGGAGATAAGG, R: GCTTCTTGCTCCCATCTTGC), *Atf4* (F: CAAAGCCCCACAACATGACC, R: CAAAGCCCCACAACATGACC), *Atf5* (F: TGGGCTGGCTCGTAGACTAT, R: TGGGCTGGCTCGTAGACTAT), and the housekeeping gene *Tbp* (F: ACCCTTCACCAATGACTCCTATG, R: TGACTGCAGCAAATCGCTTGG) were designed with the Primer-Blast tool from NCBI.

### 2.4. Western Blot

The methods for the isolation and quantification of total protein were modified from our previous study [9]. Frozen mouse liver tissue (50 mg) was homogenized in ice-cold RIPA buffer with protease and phosphatase inhibitors. The samples were then centrifuged. The protein supernatant was collected and mixed with the sample loading buffer. The mixture was then incubated in a water bath at 95–100 °C for 5 min. Equal amounts of protein from each sample were resolved on SDS–PAGE gels and transferred to a polyvinylidene difluoride membrane. The transferred membranes were blocked with 5–20 mL of 5% non-fat dry milk, followed by washing three times. Then, the membranes were incubated with primary antibodies at a 4 °C temperature with gentle shaking overnight. The primary antibodies were GCN2 (A2307, Abclonal, Wuhan, China), GCN2-p T899 (ab75836, Abcam, Cambridge, UK), eIF2α (11170-1-AP, proteintech, Wuhan, China), eIF2α-p Ser51 (28740-1-AP, proteintech), ATF5 (ab184923, Abcam, Cambridge, UK), and α-Tubulin (A6830, Abclonal). After washing three times, the membranes were incubated with secondary antibodies at room temperature for 1.5 h. Afterward, the secondary antibody was removed, and the membranes were washed three times. Detection was performed using the Thermo Scientific Supersignal™ West Pico PLUS Chemiluminescent Substrate Kit (34580, ThermoFisher Scientific).

### 2.5. Liver and Serum Lipid Measurement

Liver and serum lipids were isolated and quantified using methods from our previous study [20]. Frozen liver tissue (30 mg) was homogenized in 2 mL of ice-cold homogenization buffer (chloroform/methanol = 2:1 *v*/*v*), followed by adding 0.9% NaCl, and the mixture was vortexed and centrifuged at 2000× *g* for 10 min. The lipid fraction in the lower phase was dried with nitrogen to obtain lipid precipitation, and the lipid was solubilized with 200 µL of 30% Triton X-100. Triglyceride (TG), total cholesterol (TC), high-density lipoprotein cholesterol (HDL-c) and LDL-c levels were quantified using commercial assay kits (A110, A111, A112, and A113, Jiancheng, Nanjing, China) according to the manufacturer’s instructions. Absorbance readings were recorded using a spectrophotometer (INFINITE 200 PRO, Tecan, Männedorf, Switzerland), and lipid concentrations were calculated based on standard samples.

### 2.6. Measurement of Serum FGF21 Levels

The concentration of FGF21 in the mouse serum was measured using specific enzyme-linked immunosorbent assay (ELISA) kits (RK00368, Abclonal) [23]. Absorbance was measured using an ELISA reader (INFINITE 200 PRO, Tecan), and the serum FGF21 concentration was calculated based on the formula provided in the kit’s instructions.

### 2.7. Statistical Analysis

The statistical analyses and graphs generation were performed using GraphPad Prism software (version 10, GraphPad Software, San Diego, CA, USA) and R (version 3.5, R Foundation for Statistical Computing, Vienna, Austria). The data are expressed as the mean ± standard error of the mean (SEM). For statistical comparisons, a two-tailed Student’s *t*-test (between two groups) or one-way ANOVA with Tukey’s post hoc test (among multiple groups) was used to compare normally distributed variables. Non-normally distributed data were compared by the Mann–Whitney U test (between two groups) or the Kruskal–Wallis test (among multiple groups). *p* < 0.05 was statistically significant. A Spearman correlation analysis, assessing the relationship between *Fgf21* expression levels and host metabolite concentrations, was conducted in the R environment using the R: cor.test function.

## 3. Results

### 3.1. Gut Microbiota Contributes to KD-Induced Metabolic Phenotypes

**Figure 1:**
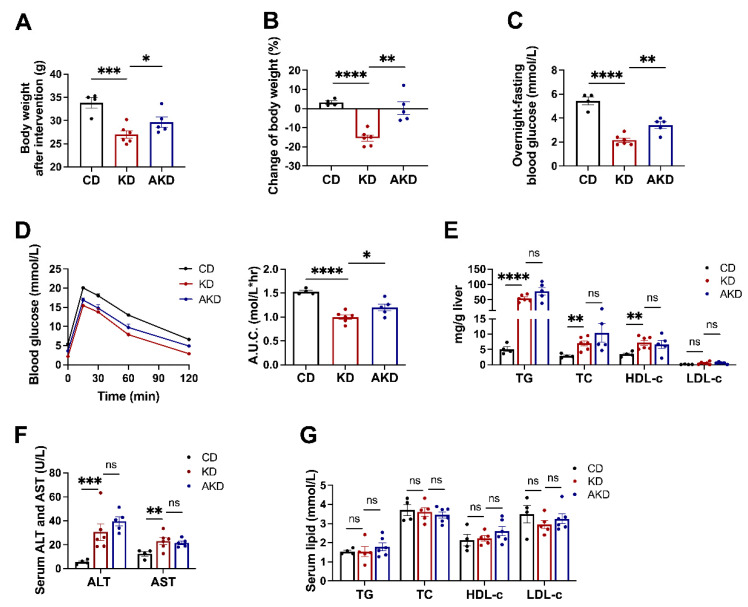
Gut microbiota significantly influences glucose metabolism in KD-fed mice. After six weeks of CD, KD, or AKD interventions on wild-type mice, we measured the following parameters for each group: (**A**) Body weight; (**B**) Percentage change in body weight; (**C**) Fasting blood glucose levels; (**D**) Glucose tolerance test results; (**E**) Liver triglyceride (TG), total cholesterol (TC), high-density lipoprotein cholesterol (HDL-c), low-density lipoprotein cholesterol (LDL-c) levels; (**F**) Serum ALT and AST levels; (**G**) Serum TG, TC, HDL-c and LDL-c levels. Statistical analysis was performed using Student’s *t*-test. “ns” indicates no significant difference; * *p* < 0.05, ** *p* < 0.01, *** *p* < 0.005, **** *p* < 0.001.

To explore the involvement of the gut microbiota in the regulation of phenotypes associated with the KD, we fed the mice with a CD, KD, and KD with antibiotic cocktail. Compared to the CD group, the KD group showed a significantly lower body weight and fasting blood glucose levels, while antibiotics intervention markedly inhibited the KD-induced changes (Figure 1A,B,D). A GTT analysis indicated that the area under the curve (AUC) in the KD group was significantly smaller than that in the CD group, whereas the AUC in the AKD group, following antibiotics treatment, rebounded significantly, becoming larger than that in the KD group. This suggests that the beneficial effects of the KD on glucose tolerance were largely diminished after gut microbiota depletion (Figure 1C). These results indicate that the gut microbiota plays a critical role in KD-induced reductions in body weight, fasting blood glucose, and improvements in glucose tolerance.

We further assessed hepatic lipid levels, serum markers of liver injury, and lipid profiles in these mice. Compared to the CD group, the KD group showed significantly higher levels of total triglycerides (TGs), total cholesterol (TC), and high-density lipoprotein cholesterol (HDL-c), along with elevated serum alanine aminotransferase (ALT) and aspartate aminotransferase (AST) levels (Figure 1E,F). However, no significant differences were observed between the KD and AKD groups in hepatic lipid levels or serum ALT and AST levels (Figure 1E,F). Additionally, no significant differences in serum lipid levels were found among the groups (Figure 1G). Therefore, we conclude that the gut microbiota is essential for KD-induced changes in body weight and blood glucose.

### 3.2. FGF21 Plays a Key Role in Microbiota-Related Metabolic Phenotypes

**Figure 2:**
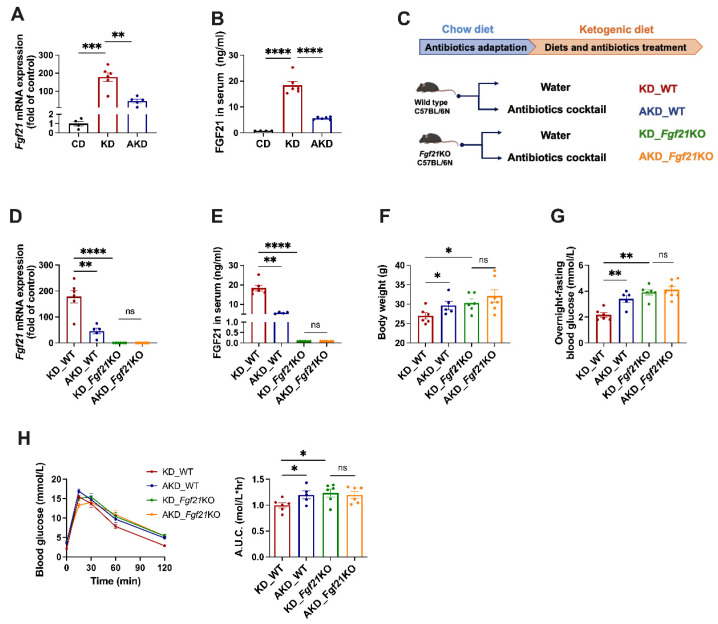
*Fgf21* is crucial for metabolic changes induced by antibiotics treatment. After six weeks of CD, KD, or AKD interventions on wild-type mice, we measured (**A**) hepatic *Fgf21* mRNA expression levels and (**B**) serum FGF21 protein levels. (**C**) Schematic of the experimental design comparing wild-type and *Fgf21*-knockout mice post-interventions. At the end of the experiment, we measured the following: (**D**) Hepatic *Fgf21* mRNA expression levels; (**E**) Serum FGF21 protein levels; (**F**) Body weight; (**G**) Fasting blood glucose; (**H**) Glucose tolerance test results. Statistical analysis was performed using Student’s *t*-test. “ns” indicates no significant difference; * *p* < 0.05, ** *p* < 0.01, *** *p* < 0.005, **** *p* < 0.001.

The next question we asked is how the gut microbiota affects body weight and glucose metabolism in the context of KDs. Previous studies have demonstrated that the KD upregulates the hepatic expression of *Fgf21*, which impacts whole-body metabolism [12,13]. We hypothesized that KD-induced changes in the gut microbiota may regulate *Fgf21* expression and function, at least partially, through Fgf21. To test this hypothesis, we measured FGF21 levels in the liver and the serum of mice from the CD, KD, and AKD groups. Hepatic *Fgf21* mRNA expression and serum FGF21 levels were significantly elevated in KD-fed mice compared to CD-fed mice (Figure 2A,B). However, after gut microbiota depletion (AKD group), hepatic *Fgf21* expression and serum FGF21 levels were significantly reduced compared to the KD group (Figure 2A,B). These results indicate that the gut microbiota can indeed regulate *Fgf21* expression.

Next, we used *Fgf21KO* mice to confirm whether *Fgf21* plays a central role in microbiota-induced metabolic changes (Figure 2C). The absence of *Fgf21* was confirmed in the *Fgf21*KO mice (Figure 2D,E). In KD-fed WT mice, antibiotics treatment significantly increased their body weight (Figure 2F) and fasting blood glucose (Figure 2G) and impaired glucose tolerance (Figure 2H). In contrast, these antibiotics-induced metabolic changes were no longer observed in *Fgf21KO* mice (Figure 2F,G), suggesting that *Fgf21* is crucial in mediating the metabolic effects of gut microbiota depletion.

### 3.3. Gut Microbiota Regulates Fgf21 Expression via the GCN2-eIF2α-ATF5 Signaling Pathway

**Figure 3:**
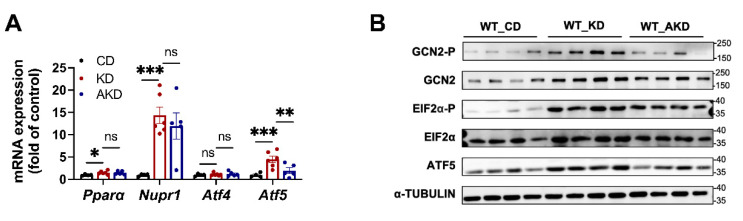
Gut microbiota in KD mice regulates *Fgf21* expression through the GCN2-EIF2α-ATF5 signaling pathway. After 6 weeks of CD, KD, or AKD interventions on wild-type mice, we measured (**A**) the hepatic mRNA expression levels of *Pparα*, *Nupr1*, *Atf4*, and *Atf5*; and (**B**) the Western blot results of the signaling molecules involved in the pathway. Statistical analysis was performed using Student’s *t*-test. “ns” indicates no significant difference; * *p* < 0.05, ** *p* < 0.01, *** *p* < 0.005

Although it has been nearly two decades since the KD was found to regulate hepatic *Fgf21* expression, the molecular mechanisms underlying this process remain unclear, especially regarding the role of the gut microbiota. Previous studies have suggested that several classic transcription factor pathways, including *Pparα*, *Nupr1*, *Atf4*, and *Atf5* [24,25], may regulate *Fgf21* expression. We examined the expression of these transcription factors in the livers of mice from the CD, KD, and AKD groups. All four were significantly upregulated in KD-fed mice, but antibiotics treatment only reduced the expression of *Atf5*, suggesting that gut microbiota may regulate *Fgf21* expression through the *Atf5* signaling pathway (Figure 3A).

The protein kinase GCN2 has been reported to activate eIF2α, with ATF5 as a downstream target of eIF2α [26]. We therefore examined the GCN2-eIF2α-ATF5 signaling pathway in the livers of the three groups. Western blot analysis revealed that the phosphorylation levels of GCN2 (GCN2-p) and eIF2α (eIF2α-p), along with ATF5 protein levels, were elevated under KD conditions (Figure 3B). Antibiotics intervention reduced the phosphorylation levels of GCN2 and eIF2α, as well as ATF5 protein levels, in the AKD group compared to the KD group. This suggests that under KD conditions, the gut microbiota may activate the GCN2-eIF2α-ATF5 pathway, which upregulates *Fgf21* expression, while gut microbiota depletion suppresses this activation (Figure 3B).

### 3.4. Dietary Changes and Antibiotics Intervention Affect Serum Metabolite Levels

**Figure 4:**
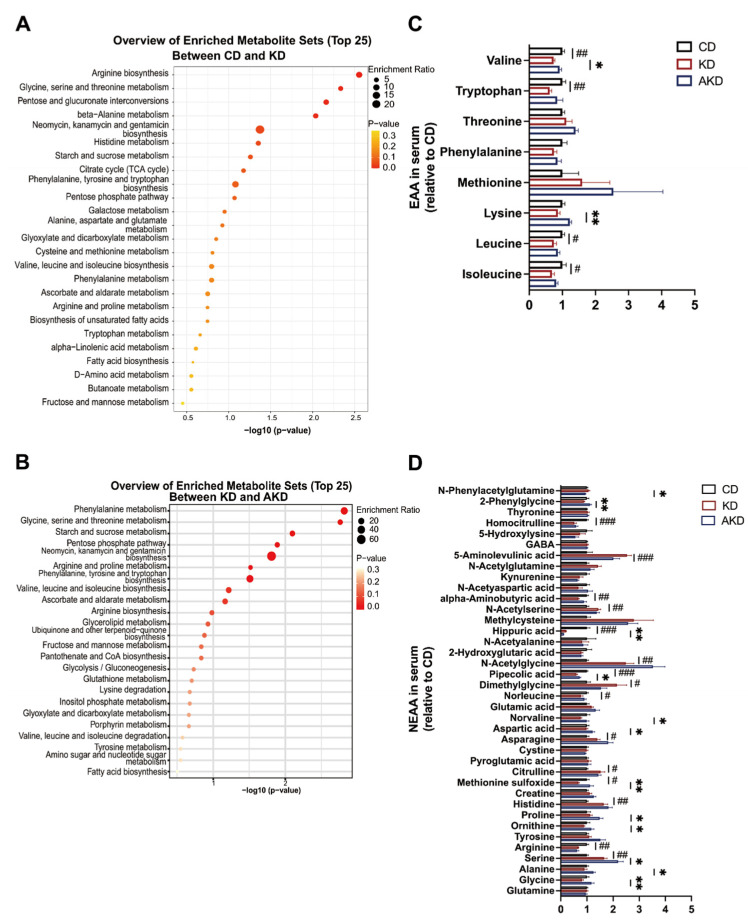
Impact of dietary changes and antibiotics treatment on serum metabolites in mice. (**A**) KEGG pathway analysis of *Fgf21*-related differential metabolites between CD and KD groups and (**B**) between KD and AKD groups. (**C**) Serum levels of essential amino acids and (**D**) non-essential amino acids across groups. Statistical analysis was performed using Student’s *t*-test. “ns” indicates no significant difference; * *p* < 0.05, ** *p* < 0.01, ^#^ *p* < 0.05, ^##^ *p* < 0.01, ^###^ *p* < 0.01.

It has been suggested that metabolites may mediate KD- and gut microbiota-induced phenotypes [18]. We collected serum from the CD, KD, and AKD groups for metabolomics analysis using the ‘Q300 Quantitative Metabolomics’ platform, which has been widely used in recent studies [27]. A comparison between the CD and KD groups revealed 79 serum metabolites significantly different between the two groups and correlated with *Fgf21* expression (|r| > 0.6, *p* < 0.05). A KEGG pathway enrichment analysis of these metabolites using the MetaboAnalyst 6.0 online tool indicated that 13 of the top 25 enriched pathways were related to amino acid metabolism or biosynthesis (Figure 4A). When comparing the KD and AKD groups, 25 metabolites were found to differ in abundance and significantly correlate with *Fgf21* expression (|r| > 0.6, *p* < 0.05), with 10 out of the 24 enriched pathways related to amino acid metabolism or biosynthesis (Figure 4B). Given that amino acid deprivation can activate protein kinases, leading to the phosphorylation of eIF2α [26,28], we hypothesized that changes in amino acids induced by the gut microbiota in KD-fed mice play a key role in regulating *Fgf21* expression.

We then performed a comparative analysis of essential and non-essential amino acids in the serum of mice from the CD, KD, and AKD groups. We screened for amino acids that exhibited significant changes between the KD and CD groups and were significantly reversed in the AKD group. Three amino acids, valine, pipecolic acid (Pip), and methionine sulfoxide (MtS), met the screening criteria. All three amino acids were downregulated in the KD group but increased after gut microbiota depletion in the AKD group (Figure 4C,D). We speculate that these amino acids were consumed excessively by the unique gut microbiota in KD-fed mice, resulting in their reduced levels in serum. Upon gut microbiota depletion, their levels increased, possibly influencing the *Fgf21* signaling pathway.

### 3.5. Valine Supplementation Regulates Fgf21 Expression and Affects Metabolism

**Figure 5:**
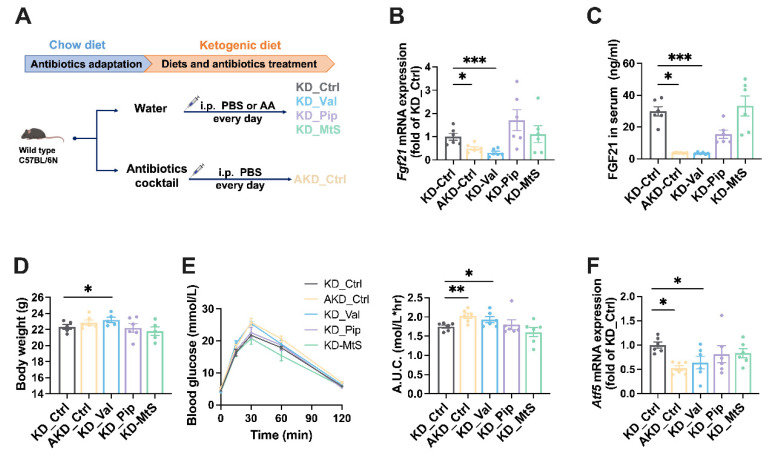
Valine supplementation affects *Fgf21* expression, body weight, and glucose tolerance in mice. Intraperitoneal supplementation of three differential amino acids in KD mice for 14 days. (**A**) Experimental schematic. At the end of the experiment, we measured the following: (**B**) hepatic *Fgf21* mRNA expression; (**C**) Serum FGF21 protein levels; (**D**) Body weight; (**E**) Glucose tolerance test results; (**F**) *Atf5* mRNA expression in the liver. Statistical analysis was conducted using Student’s *t*-test. Non label indicates no significant difference; * *p* < 0.05, ** *p* < 0.01, *** *p* < 0.005.

To investigate whether valine, Pip, and MtS affect *Fgf21* expression and metabolic phenotypes, we conducted supplementation experiments (Figure 5A). Compared to the control group, valine supplementation significantly reduced hepatic *Fgf21* mRNA expression and caused a significant increase in body weight, while Pip and MtS supplementation did not significantly affect *Fgf21* expression or body weight (Figure 5B–D). The GTT result showed that valine-supplemented mice exhibited a significantly worse glucose tolerance than the control group, while Pip and MtS had no effect (Figure 5E). Further analysis revealed that valine supplementation, unlike the other two amino acids, suppressed hepatic *Atf5* mRNA expression (Figure 5F). These results suggest that the improvement in body weight and glucose metabolism in KD-fed mice may be partially attributed to a reduction in valine levels.

## 4. Discussion

The KD is well known for its rapid weight loss effects and significant impact on glucose and lipid metabolism across various tissues and organs. However, the molecular mechanisms underlying these effects remain incompletely understood. Our study demonstrates that the gut microbiota plays a crucial role in the weight loss and changes in glucose metabolism induced by the KD. Specifically, the KD influences serum valine levels via the gut microbiota, which in turn regulates hepatic *Fgf21* expression through the GCN2-eIF2α-ATF5 signaling pathway, thereby affecting body weight and glucose metabolism in mice.

Decades ago, researchers found that KD feeding or fasting significantly increases hepatic *Fgf21* expression and circulating FGF21 levels [12,29]. Badman et al. demonstrated that fatty acids activate PPARα, leading to the upregulation of *Fgf21* [12]. More recent studies have shown that branch-chain amino acids (BCAAs) can mediate the microbiota–liver crosstalk and regulate hepatic *Fgf21* expression [30]. Our study provides the first direct evidence that the gut microbiota contributes to the KD-induced upregulation of hepatic *Fgf21* expression. Moreover, we found that the gut microbiota affects serum valine levels in mice on KDs, which contributes to the regulation of body weight and glucose metabolism through hepatic *Fgf21* expression. This finding aligns with previous studies showing that valine supplementation in both normal and high-fat diet conditions leads to increased body weight and impaired glucose metabolism [31,32]. Circulating BCAA levels impact fatty acid and glucose metabolism not only in the liver but also adipose tissue, skeletal muscle, and the heart, thereby influencing metabolic homeostasis [33,34,35]. While the GCN2-eIF2α pathway is recognized as the key signaling pathway in response to BCAA deficiency, other pathways, such as mammalian target of rapamycin complex 1 (mTORC1), AMP-activated protein kinase (AMPK), and insulin signaling, may also respond to changes in BCAA levels [34,36]. This suggests that future studies are needed to explore whether gut microbiota-mediated changes in serum valine levels in KD-fed mice may affect multiple tissues or organs and interact with various molecular signaling pathways, thereby influencing physiological phenotypes through complex crosstalk.

Recent studies suggest that certain gut microbiota, which carry genes related to amino acid metabolism, can influence the levels of amino acids in the host’s intestinal and circulatory systems [37]. This underscores the crucial role of the gut microbiota in the effects of KDs on amino acid levels. Studies have shown that certain gut microbes capable of biosynthesizing BCAAs, such as *Prevotella copri* and *Bacteroides vulgatus*, are involved in regulating circulating BCAA levels in both insulin-resistant patients and mice [38,39]. This implies that the KD may contribute to metabolic improvements by reducing circulating BCAA levels through suppressing the BCAA-producing gut microbes. In this study, we propose that gut microbes decrease serum valine levels in KD-fed mice. However, it remains unclear why KD-fed mice specifically respond to changes in valine rather than other amino acids. This specificity may be linked to the distinct gut microbial composition induced by the KD. Further investigation is required to identify the specific gut microbes involved and the mechanisms regulating serum valine levels. 16S microbiota sequencing has been widely used in recent years to study gut microbes [40], and it is intriguing to apply this method to analyze how KD-induced changes in the microbiota affect amino acid metabolism, thereby contributing to the regulation of FGF21.

Previous studies have shown that *Fgf21KO* mice exhibit an exacerbated hepatic lipid accumulation and increased levels of chylomicrons and VLDL-c when fed a KD, indicating that hepatic *Fgf21KO* aggravates abnormalities in lipid metabolism [12,41]. In our study, although gut microbiota depletion suppressed hepatic *Fgf21* expression and circulating FGF21 levels in the KD group (Figure 2A,B), there were no significant differences in lipid levels (Figure 1G). This discrepancy suggests the existence of additional pathways that rely on the gut microbiota and affect hepatic lipid metabolism in KD-fed mice, which warrant further investigation.

## 5. Conclusions

In conclusion, we explored the mechanisms by which the KD influences metabolic pathways through changes in gut microbiota composition and amino acid levels, focusing on the GCN2-eIF2α-ATF5 signaling pathway. This study reveals a complex regulatory network involving the KD, *Fgf21* expression, and gut microbiota. Future research should explore the therapeutic potential of targeting specific gut microbiota to enhance or optimize the benefits of KDs. Such approaches could pave the way for developing microbiota-modulating treatments to improve dietary interventions for metabolic disorders, including obesity and diabetes. By understanding these complex interactions, targeted strategies may emerge, offering innovative solutions for the treatment and management of metabolic diseases.

## Data Availability

The original contributions presented in the study are included in the article, further inquiries can be directed to the corresponding author.

## References

[1] Paoli A., Rubini A., Volek J.S., Grimaldi K.A. (2013). Beyond weight loss: A review of the therapeutic uses of very-low-carbohydrate (ketogenic) diets. Eur. J. Clin. Nutr..

[2] Kosinski C., Jornayvaz F.R. (2017). Effects of Ketogenic Diets on Cardiovascular Risk Factors: Evidence from Animal and Human Studies. Nutrients.

[3] Leow Z.Z.X., Guelfi K.J., Davis E.A., Jones T.W., Fournier P.A. (2018). The glycaemic benefits of a very-low-carbohydrate ketogenic diet in adults with Type 1 diabetes mellitus may be opposed by increased hypoglycaemia risk and dyslipidaemia. Diabet. Med..

[4] Zhang X., Qin J., Zhao Y., Shi J., Lan R., Gan Y., Ren H., Zhu B., Qian M., Du B. (2016). Long-term ketogenic diet contributes to glycemic control but promotes lipid accumulation and hepatic steatosis in type 2 diabetic mice. Nutr. Res..

[5] Burén J., Ericsson M., Damasceno N.R.T., Sjödin A. (2021). A Ketogenic Low-Carbohydrate High-Fat Diet Increases LDL Cholesterol in Healthy, Young, Normal-Weight Women: A Randomized Controlled Feeding Trial. Nutrients.

[6] Hansen C.D., Gram-Kampmann E.M., Hansen J.K., Hugger M.B., Madsen B.S., Jensen J.M., Olesen S., Torp N., Rasmussen D.N., Kjærgaard M. (2023). Effect of Calorie-Unrestricted Low-Carbohydrate, High-Fat Diet Versus High-Carbohydrate, Low-Fat Diet on Type 2 Diabetes and Nonalcoholic Fatty Liver Disease: A Randomized Controlled Trial. Ann. Intern. Med..

[7] Long F., Bhatti M.R., Kellenberger A., Sun W., Modica S., Höring M., Liebisch G., Krieger J.P., Wolfrum C., Challa T.D. (2023). A low-carbohydrate diet induces hepatic insulin resistance and metabolic associated fatty liver disease in mice. Mol. Metab..

[8] Nakagawa Y., Satoh A., Tezuka H., Han S.-i., Takei K., Iwasaki H., Yatoh S., Yahagi N., Suzuki H., Iwasaki Y. (2016). CREB3L3 controls fatty acid oxidation and ketogenesis in synergy with PPARα. Sci. Rep..

[9] Hong S., Moreno-Navarrete J.M., Wei X., Kikukawa Y., Tzameli I., Prasad D., Lee Y., Asara J.M., Fernandez-Real J.M., Maratos-Flier E. (2015). Nicotinamide N-methyltransferase regulates hepatic nutrient metabolism through Sirt1 protein stabilization. Nat. Med..

[10] Fernández-Rojo Manuel A., Gongora M., Fitzsimmons R.L., Martel N., Martin Sheree D., Nixon Susan J., Brooks Andrew J., Ikonomopoulou Maria P., Martin S., Lo H.P. (2013). Caveolin-1 Is Necessary for Hepatic Oxidative Lipid Metabolism: Evidence for Crosstalk between Caveolin-1 and Bile Acid Signaling. Cell Rep..

[11] Wei Z., Lei J., Shen F., Dai Y., Sun Y., Liu Y., Dai Y., Jian Z., Wang S., Chen Z. (2020). Cavin1 Deficiency Causes Disorder of Hepatic Glycogen Metabolism and Neonatal Death by Impacting Fenestrations in Liver Sinusoidal Endothelial Cells. Adv. Sci..

[12] Badman M.K., Pissios P., Kennedy A.R., Koukos G., Flier J.S., Maratos-Flier E. (2007). Hepatic fibroblast growth factor 21 is regulated by PPARalpha and is a key mediator of hepatic lipid metabolism in ketotic states. Cell Metab..

[13] Fisher F.M., Maratos-Flier E. (2016). Understanding the Physiology of FGF21. Annu. Rev. Physiol..

[14] Inagaki T., Dutchak P., Zhao G., Ding X., Gautron L., Parameswara V., Li Y., Goetz R., Mohammadi M., Esser V. (2007). Endocrine regulation of the fasting response by PPARalpha-mediated induction of fibroblast growth factor 21. Cell Metab..

[15] Laeger T., Henagan T.M., Albarado D.C., Redman L.M., Bray G.A., Noland R.C., Münzberg H., Hutson S.M., Gettys T.W., Schwartz M.W. (2014). FGF21 is an endocrine signal of protein restriction. J. Clin. Investig..

[16] Requena T., Martínez-Cuesta M.C., Peláez C. (2018). Diet and microbiota linked in health and disease. Food Funct..

[17] Wu G.D., Chen J., Hoffmann C., Bittinger K., Chen Y.Y., Keilbaugh S.A., Bewtra M., Knights D., Walters W.A., Knight R. (2011). Linking long-term dietary patterns with gut microbial enterotypes. Science.

[18] Olson C.A., Vuong H.E., Yano J.M., Liang Q.Y., Nusbaum D.J., Hsiao E.Y. (2018). The Gut Microbiota Mediates the Anti-Seizure Effects of the Ketogenic Diet. Cell.

[19] Li Y., Yang X., Zhang J., Jiang T., Zhang Z., Wang Z., Gong M., Zhao L., Zhang C. (2021). Ketogenic Diets Induced Glucose Intolerance and Lipid Accumulation in Mice with Alterations in Gut Microbiota and Metabolites. mBio.

[20] Li X., Yang J., Zhou X., Dai C., Kong M., Xie L., Liu C., Liu Y., Li D., Ma X. (2024). Ketogenic diet-induced bile acids protect against obesity through reduced calorie absorption. Nat. Metab..

[21] Fröhlich E.E., Farzi A., Mayerhofer R., Reichmann F., Jačan A., Wagner B., Zinser E., Bordag N., Magnes C., Fröhlich E. (2016). Cognitive impairment by antibiotic-induced gut dysbiosis: Analysis of gut microbiota-brain communication. Brain Behav. Immun..

[22] Secombe K.R., Ball I.A., Wignall A.D., Bateman E., Keefe D.M., Bowen J.M. (2022). Antibiotic treatment targeting gram negative bacteria prevents neratinib-induced diarrhea in rats. Neoplasia.

[23] Cao Y., Yang M., Song J., Jiang X., Xu S., Che L., Fang Z., Lin Y., Jin C., Feng B. (2023). Dietary Protein Regulates Female Estrous Cyclicity Partially via Fibroblast Growth Factor 21. Nutrients.

[24] Solon-Biet S.M., Cogger V.C., Pulpitel T., Heblinski M., Wahl D., McMahon A.C., Warren A., Durrant-Whyte J., Walters K.A., Krycer J.R. (2016). Defining the Nutritional and Metabolic Context of FGF21 Using the Geometric Framework. Cell Metab..

[25] De Sousa-Coelho A.L., Marrero P.F., Haro D. (2012). Activating transcription factor 4-dependent induction of FGF21 during amino acid deprivation. Biochem. J..

[26] Hatano M., Umemura M., Kimura N., Yamazaki T., Takeda H., Nakano H., Takahashi S., Takahashi Y. (2013). The 5′-untranslated region regulates ATF5 mRNA stability via nonsense-mediated mRNA decay in response to environmental stress. Febs. J..

[27] Deng K., Xu J.-j., Shen L., Zhao H., Gou W., Xu F., Fu Y., Jiang Z., Shuai M., Li B.-y. (2023). Comparison of fecal and blood metabolome reveals inconsistent associations of the gut microbiota with cardiometabolic diseases. Nat. Commun..

[28] Jiang H.Y., Wek R.C. (2005). Phosphorylation of the alpha-subunit of the eukaryotic initiation factor-2 (eIF2alpha) reduces protein synthesis and enhances apoptosis in response to proteasome inhibition. J. Biol. Chem..

[29] Watanabe M., Singhal G., Fisher F.M., Beck T.C., Morgan D.A., Socciarelli F., Mather M.L., Risi R., Bourke J., Rahmouni K. (2020). Liver-derived FGF21 is essential for full adaptation to ketogenic diet but does not regulate glucose homeostasis. Endocrine.

[30] Zheng H., Zhang X., Li C., Wang D., Shen Y., Lu J., Zhao L., Li X., Gao H. (2024). BCAA mediated microbiota-liver-heart crosstalk regulates diabetic cardiomyopathy via FGF21. Microbiome.

[31] Fontana L., Cummings N.E., Arriola Apelo S.I., Neuman J.C., Kasza I., Schmidt B.A., Cava E., Spelta F., Tosti V., Syed F.A. (2016). Decreased Consumption of Branched-Chain Amino Acids Improves Metabolic Health. Cell Rep..

[32] Ma Q., Hu L., Zhu J., Chen J., Wang Z., Yue Z., Qiu M., Shan A. (2020). Valine Supplementation Does Not Reduce Lipid Accumulation and Improve Insulin Sensitivity in Mice Fed High-Fat Diet. ACS Omega.

[33] Li T., Zhang Z., Kolwicz S.C., Jr Abell L., Roe N.D., Kim M., Zhou B., Cao Y., Ritterhoff J., Gu H. (2017). Defective Branched-Chain Amino Acid Catabolism Disrupts Glucose Metabolism and Sensitizes the Heart to Ischemia-Reperfusion Injury. Cell Metab..

[34] Anthony J.C., Yoshizawa F., Anthony T.G., Vary T.C., Jefferson L.S., Kimball S.R. (2000). Leucine stimulates translation initiation in skeletal muscle of postabsorptive rats via a rapamycin-sensitive pathway. J. Nutr..

[35] Green C.R., Wallace M., Divakaruni A.S., Phillips S.A., Murphy A.N., Ciaraldi T.P., Metallo C.M. (2016). Branched-chain amino acid catabolism fuels adipocyte differentiation and lipogenesis. Nat. Chem. Biol..

[36] Xiao F., Huang Z., Li H., Yu J., Wang C., Chen S., Meng Q., Cheng Y., Gao X., Li J. (2011). Leucine deprivation increases hepatic insulin sensitivity via GCN2/mTOR/S6K1 and AMPK pathways. Diabetes.

[37] Li T.T., Chen X., Huo D., Arifuzzaman M., Qiao S., Jin W.B., Shi H., Li X.V., Iliev I.D., Artis D. (2024). Microbiota metabolism of intestinal amino acids impacts host nutrient homeostasis and physiology. Cell Host Microbe.

[38] Pedersen H.K., Gudmundsdottir V., Nielsen H.B., Hyotylainen T., Nielsen T., Jensen B.A., Forslund K., Hildebrand F., Prifti E., Falony G. (2016). Human gut microbes impact host serum metabolome and insulin sensitivity. Nature.

[39] Chen H., Nie Q., Hu J., Huang X., Yin J., Nie S. (2021). Multiomics Approach to Explore the Amelioration Mechanisms of Glucomannans on the Metabolic Disorder of Type 2 Diabetic Rats. J. Agric. Food Chem..

[40] Johnson J.S., Spakowicz D.J., Hong B.Y., Petersen L.M., Demkowicz P., Chen L., Leopold S.R., Hanson B.M., Agresta H.O., Gerstein M. (2019). Evaluation of 16S rRNA gene sequencing for species and strain-level microbiome analysis. Nat. Commun..

[41] Guo W., Cao H., Shen Y., Li W., Wang W., Cheng L., Cai M., Xu F. (2024). Role of liver FGF21-KLB signaling in ketogenic diet-induced amelioration of hepatic steatosis. Nutr. Diabetes.

